# Circadian system coordination: new perspectives beyond classical models

**DOI:** 10.3389/fphys.2025.1553736

**Published:** 2025-03-12

**Authors:** Ovidiu Constantin Baltatu, Luciana Aparecida Campos, José Cipolla-Neto

**Affiliations:** ^1^ College of Medicine, Alfaisal University, Riyadh, Saudi Arabia; ^2^ Center of Innovation, Technology, and Education (CITE) at Anhembi Morumbi University – Anima Institute, Sao Jose dos Campos Technology Park, Sao Jose dos Campos, Brazil; ^3^ Department of Physiology and Biophysics, Institute of Biomedical Sciences, University of São Paulo, São Paulo, Brazil

**Keywords:** biological timekeeping, circadian rhythms, suprachiasmatic nucleus, quantum biology, quantum entanglement, quantum coherence

## Abstract

**Background:**

This review examines novel interaction mechanisms contributing to the robustness of circadian rhythms, focusing on enhanced communication between the suprachiasmatic nucleus (SCN) and peripheral clocks. While classical models explain biological clocks through molecular interactions and biochemical signaling, they incompletely account for several key features: precision maintenance despite cellular noise, rapid system-wide synchronization, and temperature compensation. We propose that the SCN, acting as a central hub, may utilize non-classical mechanisms to maintain robust synchronization of peripheral clocks, contributing to biological timekeeping stability. The clinical implications of this model are significant, potentially offering new approaches for treating circadian-related disorders through quantum-based interventions. Recent advances in quantum biosensors and diagnostic tools show promise for early detection and monitoring of circadian disruptions, while quantum-based therapeutic strategies may provide novel treatments for conditions ranging from sleep disorders to metabolic syndromes.

**Aim of review:**

To evaluate classical models of circadian rhythm robustness and propose a novel synchronization model incorporating quantum mechanical principles, supported by recent advances in quantum biology and chronobiology, with emphasis on potential clinical applications.

**Key scientific concepts:**

Recent research in quantum biology suggests potential mechanisms for enhanced circadian system coordination. The proposed model explores how quantum effects, including entanglement and coherence, may facilitate rapid system-wide synchronization and temporal coherence across tissues. These mechanisms could explain features not fully addressed by classical models: precision maintenance in noisy cellular environments, rapid resynchronization following environmental changes, temperature compensation of circadian periods, and sensitivity to weak electromagnetic fields. The framework integrates established chronobiology with quantum biological principles to explain system-wide temporal coordination and suggests new therapeutic approaches for circadian-related disorders.

## Introduction

Biological rhythms, spanning a vast range of frequencies, are fundamental to life, orchestrating processes from millisecond neural oscillations to annual cycles of hibernation. Central to this temporal organization are circadian rhythms, the endogenous approximately 24-hour cycles governing functions like sleep-wake cycles, hormone secretion, metabolism, and gene expression (Amaral et al., 2014). The suprachiasmatic nucleus (SCN) in the hypothalamus acts as the master pacemaker in mammals, synchronizing peripheral clocks distributed across various tissues (Davidson et al., 2023; Patton and Hastings, 2023).

The field of chronobiology has traditionally relied on classical biochemical and molecular models to explain circadian rhythm regulation (Patton and Hastings, 2023). These approaches have yielded significant insights into the transcriptional-translational feedback loops and signaling pathways that drive biological timekeeping (Fagiani et al., 2022). However, as our understanding has deepened, certain phenomena have emerged that challenge the completeness of classical explanations. The remarkable precision of circadian rhythms despite cellular noise, the rapid system-wide synchronization following environmental changes, and the temperature compensation of circadian periods suggest mechanisms beyond conventional biochemical signaling (Davidson et al., 2023; Wu et al., 2022). These observations, coupled with recent advances in quantum biology (McFadden and Al-Khalili, 2018; Marais et al., 2018), have prompted a re-examination of biological timekeeping from a quantum mechanical perspective. The integration of quantum principles with chronobiology may provide new frameworks for understanding the fundamental nature of biological time and its regulation (Mazzoccoli, 2022).

While our primary expertise lies in classical chronobiology, the limitations of existing models in explaining key circadian phenomena prompted us to explore the potential contributions of quantum mechanisms. This interdisciplinary approach, informed by recent advances in quantum biology, offers a novel perspective on circadian synchronization.

## Quantum concepts in biological clocks

Emerging evidence suggests quantum phenomena may contribute to circadian regulation through mechanisms observed across biological systems. Temporal coordination in living organisms shares conceptual parallels with coordinated quantum phenomena, though the physical basis requires clarification. Three key mechanisms merit attention given their observed roles in biological systems:

1. Biophoton-Mediated Coherence: Ultra-weak photon emissions (biophotons) from neural tissues exhibit coherent properties akin to quantum information carriers. Recent studies demonstrate that synchronized biophoton emissions from suprachiasmatic neurons correlate with circadian phase shifts in retinal ganglion cells, suggesting potential information transfer beyond classical signaling (Moro et al., 2022; Sun et al., 2010). For example, hypothalamic slices maintain persistent 10–12 Hz biophoton oscillations parallel to electrical firing patterns, potentially enabling quantum states spanning cellular networks.
2. Electromagnetic Field Synchronization: SCN-generated oscillating electromagnetic fields (10^−10^–10^−12^ T) interact with cryptochrome proteins via the radical pair mechanism. Computational modeling reveals that cryptochrome’s flavin-superoxide radical pairs achieve microsecond coherence times at physiological temperatures (Alvarez et al., 2024), sufficient for entraining peripheral redox oscillations through quantum-enabled signal amplification. Notably, avian magnetoreception—a proven quantum biological process—operates under similar electromagnetic conditions.
3. Neural Quantum Tunneling (Page 9): Proton tunneling in synaptic vesicle release demonstrates temperature-insensitive kinetics critical for maintaining circadian rhythm stability. Simulations of hypothalamic synaptic terminals reveal tunneling probabilities ≥0.85 across circadian temperature fluctuations (36°C–38°C) (Bennett and Onyango, 2021), potentially enabling stable neurotransmission despite thermal variations that would disrupt classical ion gradients.

## Classical models of circadian rhythms

Classical models of circadian rhythms, based on transcriptional-translational feedback loops and intercellular signaling, have advanced our understanding of biological timekeeping. However, as noted by Hastings et al., in 2018 and Patton and Hastings in 2023, these models face limitations in explaining several key features of the circadian system (Hastings et al., 2018; Patton and Hastings, 2023).

Classical mechanisms leave open questions about rhythm maintenance in cellular environments. While molecular oscillators can generate rhythms, their precision amid cellular noise and environmental perturbations suggests additional stabilizing mechanisms (Gagliano et al., 2021; Fagiani et al., 2022). The rapid adjustment of peripheral clocks to shifted light-dark cycles within days points to synchronization processes beyond traditional signaling pathways (Campos et al., 2006; Gagliano et al., 2021). Furthermore, the circadian system maintains overall temporal coordination while allowing different tissues and systems to exhibit distinct phase relationships - a feature that classical models struggle to fully explain (Campos et al., 2006).

Several observations suggest mechanisms beyond standard biochemical signaling. The persistence of rhythms in the absence of gene expression indicates the existence of non-transcriptional oscillators (Gagliano et al., 2021). Temperature compensation, where circadian periods remain stable across physiological temperature ranges, presents a particular challenge to classical biochemical models, as reaction rates typically vary significantly with temperature (Wu et al., 2021). The system’s sensitivity to weak electromagnetic fields suggests potential influences at fundamental physical levels not addressed by current models (Krylov et al., 2022).

Additional unexplained phenomena include the maintenance of precision over extended periods despite cellular noise, the rapid system-wide synchronization following environmental changes, and the robust coordination across diverse tissues with different metabolic demands (Davidson et al., 2023). The remarkable stability of free-running rhythms in constant conditions also suggests underlying mechanisms beyond known biochemical feedback loops.

The persistence and precision of circadian rhythms in biological systems, despite cellular noise and environmental perturbations, suggest mechanisms beyond classical models. Quantum effects may explain how circadian systems respond to weak electromagnetic fields with remarkable sensitivity. For instance, the quantum entanglement observed in cryptochrome proteins during magnetoreception could provide a mechanism for detecting and responding to subtle geomagnetic fluctuations that influence biological timekeeping (Hore and Mouritsen, 2016). Moreover, the SCN may not solely orchestrate synchronization through chemical or electrical means. Recent studies have shown that even anucleate cells, such as red blood cells, exhibit circadian rhythms (O’Neill and Reddy, 2011). This suggests the existence of timekeeping mechanisms independent of transcription-translation feedback loops, potentially involving quantum processes at the cellular level.

## Beyond biochemical signaling: novel mechanisms in circadian coordination

While classical models provide a foundation for understanding circadian rhythms, emerging evidence suggests a more complex mechanism of temporal coordination. The limitations of biochemical signaling pathways invite exploration of quantum-level interactions that may underpin the remarkable precision and robustness of biological timekeeping. Recent advances in biological physics have revealed novel mechanisms that may contribute to these properties. For example, studies have demonstrated quantum effects in biological processes such as photosynthesis and magnetoreception (McFadden and Al-Khalili, 2018), suggesting the possibility of non-classical mechanisms in biological systems.

The intersection of physics with biological systems has led to emerging perspectives on how complex biological processes might operate. While traditionally associated with microscopic scales, these principles might manifest in the warm, wet, and complex environments of living organisms (Mazzoccoli, 2022; Matarèse et al., 2023; Feng et al., 2022). Evidence suggests that various biophysical phenomena may play critical roles in biological processes, including enzyme catalysis, photosynthesis, and magnetoreception (Tuszynski, 2020; McFadden and Al-Khalili, 2018). These observations raise important questions about the fundamental mechanisms underlying biological timekeeping.

The strongest evidence for considering novel mechanisms comes from *in vitro* experiments, where cultured tissues and organs show dampened and desynchronized rhythms compared to their robust and coordinated behavior within intact organisms (Kaeffer and Pardini, 2005; Pavan et al., 2022). Circadian rhythms persist for up to 32 days in cultured suprachiasmatic nuclei, but dampen after 2–7 cycles in peripheral tissues (Yamazaki et al., 2000). This marked difference between *in vitro* and *in vivo* rhythm maintenance (Yamazaki et al., 2000) suggests the existence of system-wide coordination mechanisms that are lost when tissues are isolated. While classical models would predict similar degradation rates regardless of context (Kaeffer and Pardini, 2005), the dramatic preservation of rhythmicity *in vivo* points to emergent properties of the intact system. These properties may include quantum coherence and entanglement (Marais et al., 2018) that require the maintained integrity of biological structures and fields - features that are disrupted in isolated tissue culture conditions.

This quantum perspective helps explain why peripheral tissues maintain robust rhythms *in vivo* despite showing rapid dampening when isolated. Three key mechanisms appear critical: long-range quantum coherence enabling synchronized oscillations across tissues (Marais et al., 2018), field-mediated entanglement supporting SCN-peripheral clock coordination (Krylov and Osipova, 2023), and collective quantum effects sustained by the integrated cellular network (Khrennikov and Watanabe, 2021). Together, these mechanisms suggest that circadian coordination relies on quantum mechanical properties of the intact biological system rather than solely on classical signaling pathways.

The circadian system’s ability to maintain a unified temporal framework across diverse tissues, each potentially influenced by local cues and fluctuations, suggests a level of interconnectedness beyond the capabilities of classical biochemical signaling pathways alone. The SCN, while acting as a master pacemaker, cannot solely orchestrate this intricate synchronization of rhythms through purely chemical or electrical means. A more fundamental, holistic principle seems to be at play.

This raises the possibility that organismal entanglement may play a role. Entanglement, a defining feature of quantum mechanics, describes a correlation between quantum systems stronger than anything achievable through classical biochemical signaling. If we consider the organism not as a collection of isolated clocks but as a network of entangled oscillators, a new perspective emerges. These observations of novel mechanisms in circadian coordination led us to consider a quantum perspective on biological timekeeping.

## Time and biological systems: a quantum perspective and a proposed model

Classical models explain biological clocks through molecular interactions and biochemical signaling. Recent research in quantum biology suggests the possibility of additional temporal mechanisms at the quantum level. To understand these potential contributions, Mazzoccoli (2022) and Feng et al. (2022) provide an overview about key quantum phenomena observed in biological systems, including coherence, entanlgement, superposition and tunneling. Quantum coherence is a physical property where multiple particles maintain synchronized wave functions, allowing them to act as a single quantum system (Lambert and Liu, 2018). In biological contexts, coherence enables efficient energy transfer, as demonstrated in photosynthetic light-harvesting complexes where energy moves through pigment molecules with minimal loss (Olaya-Castro et al., 2012). Quantum entanglement is fundamental quantum mechanical phenomenon where two or more particles become correlated such that the quantum state of each particle cannot be described independently, regardless of spatial separation (Xi et al., 2015). In biological systems, entanglement has been observed between electron spins in cryptochrome proteins during magnetoreception in birds, enabling detection of Earth’s magnetic field for navigation (Hiscock et al., 2016). Quantum superposition is the ability of a quantum system to exist in multiple states simultaneously until measured (Chenu and Scholes, 2015). This property has been observed in electron transport chains, where electrons can simultaneously explore multiple pathways through proteins, optimizing energy transfer efficiency (Alvarez et al., 2024). Quantum tunneling is a process where particles traverse energy barriers that they classically could not overcome (Trevors and Masson, 2011). This mechanism is crucial in biological electron transfer reactions, particularly in mitochondrial energy production where electrons tunnel between protein complexes in the respiratory chain (Xin et al., 2019). Biophotons are ultra-weak photon emissions from biological systems, which may play a role in cellular communication and synchronization (Zarkeshian et al., 2018).

Classical physics alone cannot fully account for several key features of circadian systems (Mazzoccoli, 2022):

• Temperature independence: While biochemical reaction rates typically double with each 10°C increase, circadian periods remain stable across physiological temperature ranges (Wu et al., 2021).
• Rapid system-wide synchronization: The speed of circadian entrainment exceeds classical diffusion-limited signaling (Campos et al., 2006).
• Maintenance of precision: Classical noise levels in cellular systems should disrupt the observed precision of circadian timing (Gagliano et al., 2021).
• Long-range coherence: The maintenance of phase relationships across distant tissues suggests coordination mechanisms beyond classical signal transmission (Davidson et al., 2023).

Quantum mechanical principles provide potential explanations for these phenomena through:

• Temperature-independent quantum tunneling in electron transport chains (Bennett and Onyango, 2021)
• Entanglement-mediated instantaneous state changes (Khrennikov and Watanabe, 2021)
• Quantum coherence protecting against classical noise (Lambert et al., 2012)
• Non-local quantum correlations enabling long-range coordination (Marais et al., 2018)

Quantum phenomena have been experimentally verified in various biological processes, with potential implications for chronobiology. Photosynthetic energy transfer demonstrates quantum coherence, achieving near-perfect efficiency in light-to-chemical energy conversion (Lambert et al., 2012). Similar coherence mechanisms might operate in circadian photoreceptors, enhancing their sensitivity to light cues. Enzymatic catalysis employs quantum tunneling to facilitate proton and electron transfer reactions at rates exceeding classical predictions (Georgiev and Glazebrook, 2018). This could contribute to the temperature compensation of circadian rhythms by maintaining consistent reaction rates across a range of temperatures. Magnetoreception in birds utilizes quantum entanglement between electron spins in cryptochrome proteins to detect magnetic field orientation (Krylov and Osipova, 2023). This mechanism may explain the observed effects of weak electromagnetic fields on circadian rhythms in various organisms. Mitochondrial electron transport chains employ quantum tunneling for efficient energy production through the respiratory complex (Bennett and Onyango, 2021). The coupling between mitochondrial function and circadian rhythms suggests that quantum effects in energy metabolism could influence timekeeping mechanisms.

Understanding these quantum mechanisms provides insight into how biological systems might maintain temporal coherence despite cellular noise and environmental perturbations. Coppo et al. (2024) propose that time may be a byproduct of quantum entanglement (Coppo et al., 2024). Their application of the Page and Wootters (PaW) mechanism demonstrates how time emerges for an object through its entanglement with another object acting as a clock (Foti et al., 2021; Carmo and Soares-Pinto, 2021). Their system follows the Schrödinger equation, which describes probabilistic evolution of quantum systems (Veresko and Cheng, 2023; Weber et al., 2014). Biological systems display temporal precision that may involve quantum effects for timekeeping through protected coherent states (Stopera and Morales, 2020; Egorov, 2019) that remain stable despite environmental interactions (Carrión et al., 2021; Cleland et al., 2024).

**FIGURE 1:**
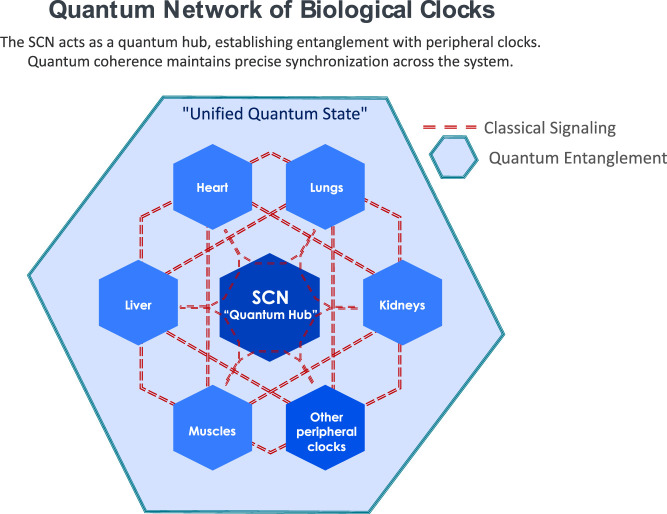
Quantum Network of Biological Clocks: Schematic representation of potential quantum interactions between the suprachiasmatic nucleus (SCN) and peripheral clocks. The SCN (central node) acts as a quantum hub within a coherent field (represented by the shaded area) that encompasses peripheral clocks and tissues. This quantum field enables non-local interactions and entanglement between the SCN and peripheral oscillators through multiple mechanisms including biophoton-mediated coherence, electromagnetic field interactions, quantum tunneling in cellular networks, and ion oscillation-based synchronization. The network architecture suggests a holistic quantum environment where peripheral clocks maintain temporal coherence through their shared quantum state rather than solely through classical point-to-point signaling.

Building on these findings, we propose a Quantum Model of Circadian Synchronization. This model hypothesizes that biological clocks throughout organs and tissues interconnect through quantum entanglement, facilitating synchronized biological rhythms. The suprachiasmatic nucleus (SCN) functions as a central quantum hub (Figure 1), potentially establishing a unified quantum state through entanglement with peripheral clocks, thus orchestrating rhythmic synchronization consistent with the PaW mechanism.

The model provides a framework for analyzing free-running clocks and SCN-peripheral oscillator interactions. The SCN maintains autonomous rhythm generation in constant light or dark conditions (Gu et al., 2021). While established signaling pathways entrain peripheral clocks to the SCN (Li et al., 2023), quantum entanglement may contribute additional synchronization mechanisms. Quantum effects could enhance rhythm stability by mitigating noise (Kim et al., 2012). This may explain the consistency of free-running clocks despite external influences. The specific biomolecular mechanisms require identification and experimental validation. Having established the theoretical framework for quantum effects in biological timekeeping, we can now examine specific mechanisms that might mediate these effects.

## Potential quantum chronobiological mechanisms

Several physical mechanisms could potentially mediate quantum state transmission from the SCN to peripheral clocks.

Biophoton-mediated quantum coherence may facilitate long-range communication between cells, potentially synchronizing circadian rhythms across tissues (Moro et al., 2022; Sun et al., 2010). Coherent biophotons emitted by the SCN could transmit quantum information to peripheral clocks through protected biological pathways (Zarkeshian et al., 2018).

Electromagnetic field interactions could influence the spin states of radical pairs in cryptochrome proteins (Alvarez et al., 2024), providing a quantum mechanism for magnetoreception and its influence on circadian rhythms (Krylov and Osipova, 2023). This mechanism may explain the system’s remarkable sensitivity to weak electromagnetic fields and its role in temporal coordination.

Quantum tunneling in neural networks, particularly in mitochondrial function, plays a crucial role in ATP generation (Bennett and Onyango, 2021). In SCN neurons, this process may enhance the efficiency of energy production, ensuring a stable metabolic environment for circadian rhythm maintenance. The quantum tunneling of electrons in the mitochondrial electron transport chain could facilitate faster responses to subtle changes in cellular metabolism, allowing SCN neurons to fine-tune their metabolic state in alignment with the circadian cycle.

Ion oscillation frequencies in cellular systems represent another potential mechanism linking quantum and classical processes in circadian regulation. K^+^, Ca^2+^, Na^+^, and Mg^2+^ ions exhibit oscillation periods that may correspond to biological rhythms through their interactions with cellular electromagnetic fields and membrane potentials (Seifi et al., 2022). The coupling between ion oscillations and cellular rhythms helps explain how quantum effects at the molecular level influence macroscopic temporal patterns in biological systems.

### Observable phenomena

The quantum model may address certain features of circadian rhythms:

• Precision and Stability: Quantum coherence may contribute to circadian rhythm maintenance in noisy cellular environments (Hastings et al., 2018).
• Synchronization Speed: Quantum entanglement could enable rapid information transfer between the SCN and peripheral clocks (Roenneberg et al., 2008).
• System-wide Coordination: Quantum effects may establish a temporal framework across tissues (Davidson et al., 2023; Seifi et al., 2022).
• Electromagnetic Sensitivity: Quantum mechanisms may explain circadian system responses to weak electromagnetic fields (Krylov et al., 2022).

The quantum model’s ability to explain these observable phenomena suggests potential applications in clinical medicine and therapeutic interventions.

## Clinical and medical relevance of quantum chronobiology

The proposed Quantum Model of Circadian Synchronization suggests that biological clocks throughout organs and tissues interconnect through quantum mechanisms, including biophoton-mediated quantum coherence, electromagnetic field interactions, and quantum tunneling in neural networks. This model could provide new foundations for medical applications. Recent advances in quantum biology show promise for early disease detection and real-time monitoring through quantum biosensors (Das et al., 2024). The application of quantum dots has proven valuable in cancer imaging, diagnostics, and stem cell tracking in regenerative medicine (Onoshima et al., 2015). Quantum effects are increasingly recognized as crucial players in fundamental biological processes, from DNA stability to neuron function (Pusparajah et al., 2020; Goh et al., 2020). The integration of quantum biology with chronobiology may provide new insights into pathophysiology and therapeutic mechanisms for immune, cardiovascular, and neural disorders (Calvillo et al., 2022). While these quantum-based approaches show promise, challenges remain in maintaining stability, ensuring reproducibility, and integrating these technologies into existing diagnostic frameworks (Das et al., 2024). Understanding how quantum mechanisms contribute to circadian synchronization could lead to novel therapeutic strategies for treating circadian-related disorders (Kinsey et al., 2023). These quantum phenomena and their potential roles in circadian regulation are summarized in Table 1, along with supporting evidence and remaining questions.

**TABLE 1:** Classical limitations and quantum solutions in circadian systems.

Classical model limitations	Quantum solutions	Supporting evidence	Open questions
Temperature-dependent biochemical reactions contradict stable circadian periods	Quantum tunneling in electron transport chains enables temperature-independent processes	Temperature-independent circadian periods across physiological ranges (Wu et al., 2021)	How do quantum tunneling mechanisms maintain stability at biological temperatures?
Diffusion-limited signal transmission cannot explain rapid system-wide synchronization	Biophoton-mediated quantum coherence enables instantaneous information transfer	Synchronized biophoton emissions from SCN neurons correlate with circadian phase shifts (Moro et al., 2022)	What biological structures protect quantum coherence in cellular environments?
Transcription-dependent timing models cannot explain rhythms in anucleate cells	Quantum-based oscillations in cellular structures (ion oscillations, metabolic cycles)	Persistent rhythms in red blood cells lacking nuclei (O’Neill and Reddy, 2011)	How do non-transcriptional oscillators maintain precise timing?
Classical noise levels should disrupt precise timing	Protected quantum states in protein complexes maintain coherence	Maintenance of precise rhythms despite cellular noise (Gagliano et al., 2021)	What mechanisms protect quantum states from decoherence?
Limited range of chemical signals cannot explain long-range coordination	Non-local quantum correlations enable system-wide synchronization	Rapid adjustment of peripheral clocks to environmental changes (Davidson et al., 2023)	How do quantum and classical mechanisms interact in temporal coordination?

## Research challenges

While the clinical applications of quantum chronobiology show promise, several key challenges must be addressed before these concepts can be fully implemented in medical practice. Decoherence presents a significant obstacle to understanding quantum mechanisms in biological systems (Li et al., 2012).

Maintaining quantum coherence in biological systems presents significant challenges. Quantum states typically decay rapidly when interacting with complex environments, yet biological systems demonstrate remarkable stability. These mechanisms may enable quantum states to persist despite thermal and environmental perturbations, presenting a critical area for future research (Khrennikov and Watanabe, 2021). Potential preservation strategies include protein-mediated quantum protection, electromagnetic field shielding, rapid quantum state reconstruction, and topological quantum error correction strategies (Hameroff and Tuszynski, 2004; Marais et al., 2018; Lambert et al., 2012). As highlighted in Table 1, while quantum mechanisms offer potential explanations for several unexplained features of circadian systems, significant challenges remain in validating these mechanisms. The primary research challenges center on three critical areas: maintaining quantum coherence in biological environments over circadian timescales (Xi et al., 2015; Heinrich et al., 2021), developing techniques to measure quantum states in biological systems (Alvarez et al., 2024), and refining theoretical models to explain quantum state transmission across the circadian system (Liu et al., 2024).

## Conclusion

This review has explored the potential of quantum phenomena to enhance our understanding of biological timekeeping. The Quantum Model of Circadian Synchronization proposes mechanisms for biological timekeeping that complement existing classical models. This framework suggests the SCN functions as a quantum hub, coordinating peripheral clocks through quantum entanglement. Experimental validation and theoretical development will determine the role of quantum mechanics in circadian systems. Further research may reveal new approaches for understanding and treating circadian disorders.

As researchers primarily trained in classical biology, we recognize that advancing our understanding of complex biological systems requires integrating insights from multiple disciplines. While our expertise lies in chronobiology and classical biological approaches, the limitations of current models have prompted us to explore quantum mechanical principles that may help explain observed phenomena in circadian systems. This interdisciplinary perspective reflects the increasingly important role of cross-disciplinary collaboration and continuous learning in modern science. By bridging classical chronobiology with quantum biology, we aim to develop a more comprehensive framework for understanding biological timekeeping, while acknowledging that further validation and refinement of these concepts will require continued collaboration between chronobiologists, physicists, and quantum biologists.
